# Supplementary material for: Unexpected Differences in the Speed of Non-Malignant versus Malignant Cell Migration Reveal Differential Basal Intracellular ATP Levels
Source: Cancers (Basel). 2023 Nov 22;15(23):5519. doi: 10.3390/cancers15235519 (PMC10705159; doi:10.3390/cancers15235519)
Supplement: Supplementary file 1 [file cancers-15-05519-s001.zip › cancers-2718792-supplementary.pptx]

## Slide 1
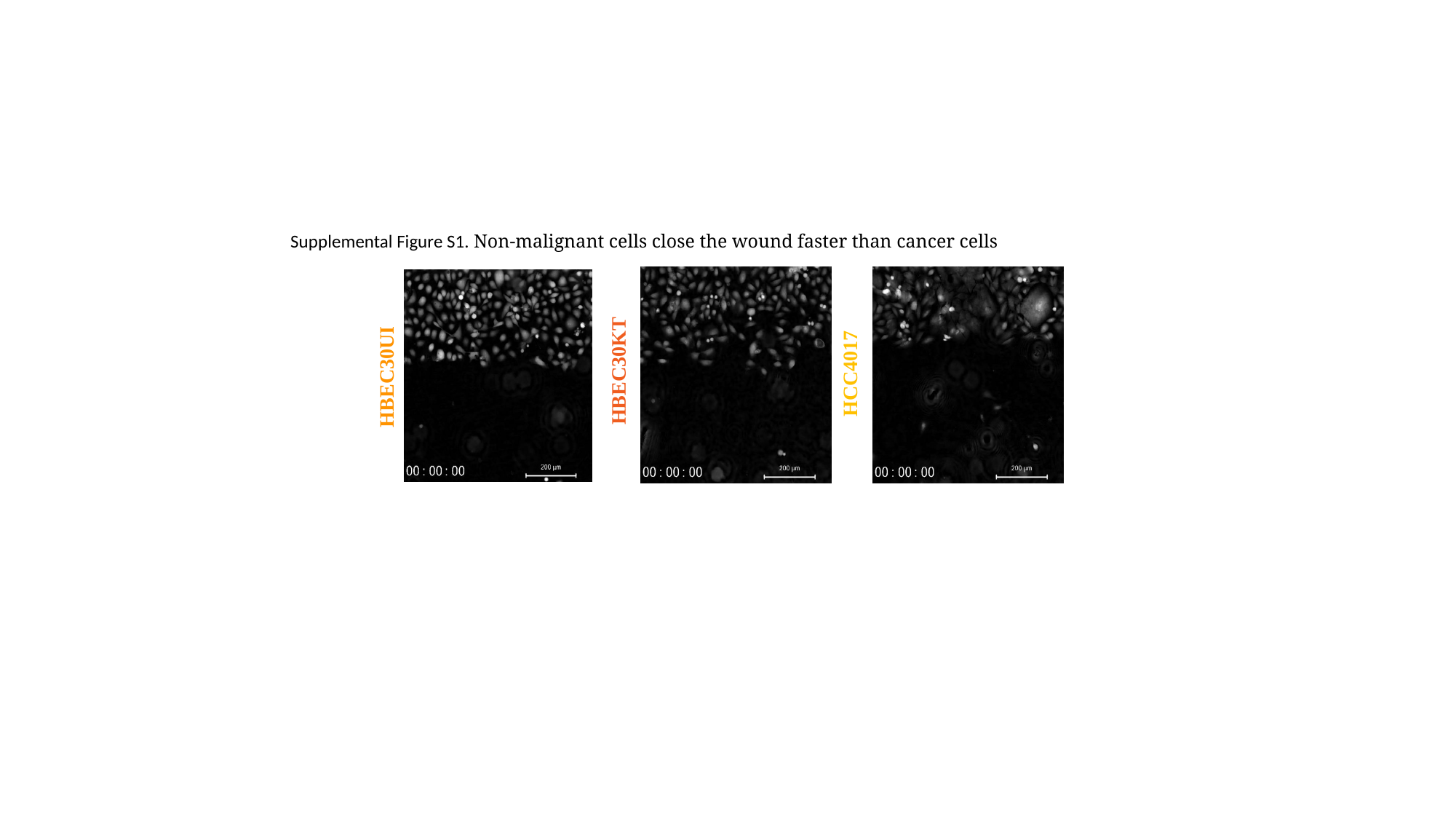

Supplemental Figure S1. Non-malignant cells close the wound faster than cancer cells
HBEC30KT
HBEC30UI
HCC4017
